# Green Fabrication and Release Mechanisms of pH-Sensitive Chitosan–Ibuprofen Aerogels for Controlled Transdermal Delivery of Ibuprofen

**DOI:** 10.3389/fchem.2021.767923

**Published:** 2021-11-08

**Authors:** Chen Li, Ke Wang, Dong Xie

**Affiliations:** ^1^ Institute of Biological and Medical Engineering, Guangdong Academy of Sciences, Guangzhou, China; ^2^ Guangdong Biomaterials Engineering Technology Research Center, Guangzhou, China

**Keywords:** transdermal drug delivery system, chitosan–ibuprofen aerogels, ex vivo skin permeation, drug release mechanism, pH-responsive

## Abstract

Ibuprofen is a potent non-steroidal anti-inflammatory drug due to its analgesic, antipyretic, and anti-inflammatory actions. However, its poor solubility in water makes it difficult to manufacture ibuprofen tablets, which limited the application of ibuprofen in drug delivery systems. Polymer–drug aerogels have attracted huge interest in optimizing the drug delivery efficiency and improving the physicochemical characteristics and therapeutic quality. Here, chitosan–ibuprofen aerogels with excellent swelling, high biocompatibility, and better drug delivery efficiency were synthesized by a simple method. Our study found that the chitosan–ibuprofen aerogels exhibited remarkably improved thermal stability, excellent swelling ratio, and high drug loading. As a consequence of these favorable properties, the chitosan–ibuprofen aerogels exhibited improved drug delivery efficiency and achieved drug prolonged administration. Our study highlights the great potential of polymer–drug aerogels in improving the drug delivery efficiency of transdermal drug delivery systems.

## 1 Introduction

With the improvement of life quality, the irregular lifestyle of people results in the development of chronic diseases such as osteoarthritis, diabetes, hypertension, low back pain, neck pain, Parkinson’s disease, and rheumatoid arthritis (Chiang et al., 2021). It is well known that ibuprofen is an effective non-steroidal anti-inflammatory drug used for the management of pain, symptoms of rheumatoid arthritis, and fever. However, its low solubility, short biological half-life, and rapid clearance after oral administration limited the wide application and lead to wasted dosing and potentially serious side effects (Wang et al., 2020a; Uchiyama et al., 2021; Ćirić et al., 2021). Traditional drug delivery systems cannot satisfy the long-term and continuous release of ibuprofen. In recent years, researchers found that the transdermal drug delivery system can provide long-term and sustained drug delivery, and it is a promising drug delivery system in the treatment of these chronic diseases (Choudhary and Singh, 2021; Bose et al., 2021; Anantrao et al., 2021; Hanumanaik* et al., 2012). The transdermal drug delivery system works very simply, and it has become an important field due to its self-advantages such as avoidance of first-pass metabolism, stable and controlled blood level, ease of termination of drug action, long duration of actions, and no interference with gastric and intestinal fluid (Chu et al., 2020; Fukuta et al., 2020; Hu et al., 2020; Sabri et al., 2020; Yang et al., 2020).

However, the development of transdermal formulations for ibuprofen is a complex work because of its short biological half-life, low solubility, and intrinsically poor skin permeability (Abioye et al., 2015; Morrison et al., 2021; Theochari et al., 2021). Therefore, developing novel transdermal formulations of ibuprofen has attracted great interest to reduce the dosing waste and the side effects (Kashyap et al., 2020; Xia et al., 2020; Yadav et al., 2021). Campardelli et al. prepared porous polycaprolactone patches impregnated with nimesulide by supercritical carbon dioxide. They were used in transdermal drug delivery systems for reducing the number of drug administrations (Campardelli et al., 2019). Polymer aerogels are also the promising material in the design of transdermal delivery systems for ibuprofen (Wang et al., 2020b; Ramöller et al., 2020; Jaber et al., 2021), and they can achieve a suitable combination of polymer–drug compatibility, skin permeation, and *in vitro* release kinetics and swelling ratio. However, it is the major challenge to develop the ibuprofen formulations for controlling the release of drugs in transdermal delivery systems and achieving drug prolonged administration (Wang et al., 2020b; Ćirić et al., 2021).

Chitosan derived from chitin is a linear cationic poly(b-(1-4)-2-amino-2-deoxy-D-glucan). It is a potential polymer to be used for preparing transdermal drug delivery systems (TDDSs) due to its abundant natural occurrence, biocompatibility, good mechanical properties, bioadhesive properties, biodegradability, low toxicity, and enhancer absorption effect (El Menshawe et al., 2020; Castilla-Casadiego et al., 2021; Morad et al., 2021; Takeuchi et al., 2021; Talib et al., 2021). Among these chitosan-based TDDSs, chitosan–ibuprofen aerogel has attracted more and more attention because of its excellent swelling ratio and ease of preparation, and in addition, it can open the tight junction in chitosan–ibuprofen to improve the drug penetration through mucosal tissues.

Herein, we prepared chitosan–ibuprofen aerogels by a simple method for controlled release of ibuprofen. Their morphology, microstructure, and phase structure were evaluated by scanning electron microscopy (SEM), Fourier-transform infrared (FTIR) spectrometry, UV-Vis spectroscopy, and X-ray diffraction (XRD). The thermal properties of chitosan–ibuprofen aerogels were determined using differential scanning calorimetry (DSC) and thermogravimetric analysis (TGA). As a consequence, the as-prepared chitosan–ibuprofen aerogels exhibited remarkably improved thermal stability and excellent swelling ratio and achieved drug prolonged administration. This work may be a promising method to design a polymer–drug transdermal delivery system for controlling the release of ibuprofen and other poorly water-soluble drugs.

## 2 Experimental Section

### 2.1 Materials and Chemicals

Ibuprofen (C_13_H_18_O_2_, ≥98%), chitosan (degree of deacetylation ≥95%, viscosity 100–200 MPa.s), sodium hydroxide (NaOH, 96%), glacial acetic acid (CH_3_COOH, ≥99.5%), dialysis bag (MWCO, 3,000 Da), potassium phosphate monobasic (KH_2_PO_4_, 99.5%), and ethanol (C_2_H_5_OH, 99%) were all purchased from commercial sources. Deionized (DI) water was used throughout the experimental processes. All other chemicals used in this work were of analytical grade.

### 2.2 Preparation of Chitosan–Ibuprofen Aerogels

Chitosan–ibuprofen aerogels were prepared using a simple method slightly performed according to the previously reported method (Abioye et al., 2015). In brief, chitosan solutions were prepared by dissolving chitosan powder in 10 ml of 1% glacial acetic acid at 45°C, and ibuprofen was solubilized in 10 ml of 0.1M NaOH solution. Then, the two obtained solutions were made up to 25 ml with distilled water, separately. Finally, the ibuprofen solution was added into the chitosan solution drop by drop and allowed to react for 2 h at 45°C under continuous stirring. The colloidal dispersion was vacuum-filtrated by a water-circulation multifunction vacuum pump with a vacuum filter holder. Then, they were kept in a refrigerator at −4°C and used for lyophilizing at −20 °C under vacuum to obtain the target sample.

### 2.3 Characterization of Chitosan–Ibuprofen Aerogels

FTIR spectra of the samples were investigated via an FTIR spectrophotometer (INVENIO-S) over wavenumbers ranging from 4,000 to 400 cm^−1^ with 0.5 cm^−1^ resolution using KBr pellets. UV-Vis spectroscopy was performed over wavelengths from about 200 nm to about 400 nm *via* a UV-Vis spectrophotometer (TU 1901). X-ray diffraction (XRD, X'Pert PRO Ultima IV) patterns of the samples were recorded at a scan rate of 2°/min in the scan range of 2θ from 10° to 80° with an X-ray diffractometer. The field-emission scanning electron microscope (FE-SEM, ZEISS MERLIN) was used to evaluate the morphology and microstructure of ibuprofen, chitosan, and chitosan–ibuprofen aerogel samples.

### 2.4 Thermal Behavior of Chitosan**–**Ibuprofen Aerogels

The thermal properties of ibuprofen, chitosan, and chitosan**–**ibuprofen aerogels were determined via differential scanning calorimetry (DSC, DSC 25) and thermogravimetric analysis (TGA, TG209 F3). 5–8 mg of the sample was placed in an aluminum pan and heated at temperatures between 0°C and 200°C at a heating rate of 20°C/min under nitrogen with a flow of 20 ml/min. The thermogravimetric analysis was evaluated with a thermogravimetric analyzer (PerkinElmer Ltd., Beaconsfield, United Kingdom). 5–10 mg of the sample was placed in an alumina pan and put into crucible baskets at temperatures ranging from 25°C to 800°C at a heating rate of 20°C/min.

### 2.5 Drug Loading Efficiency

The drug loading efficiency of chitosan**–**ibuprofen aerogels was determined by a previously reported method with slight modifications. 20 mg of chitosan**–**ibuprofen aerogels was placed in 10 ml of PBS solution of pH 7.4 at 37°C for 6 h. The ibuprofen concentration was measured by a UV-Vis spectrophotometer at 265 nm. The drug loading efficiency of chitosan**–**ibuprofen aerogels was calculated by

$$\text{Loading}\;\text{efficiency}\left(\%\right)=\frac{M_{1}}{M_{0}}\times 100\%,$$ (1)

where M_1_ is the amount of ibuprofen in chitosan**–**ibuprofen aerogels and M_0_ is the chitosan**–**ibuprofen aerogels.

### 2.6 Swelling Degree Analysis

The swelling ratio of chitosan**–**ibuprofen aerogels was measured in various types of electrolytes at room temperature. In brief, 0.5 g of chitosan and chitosan**–**ibuprofen aerogel samples was immersed in deionized water in a shaking water bath at 37°C for 60 h to reach swelling equilibrium. At fixed time intervals, the swollen chitosan and chitosan–ibuprofen nanoconjugate samples were weighed after wiping the floating water by a filter paper. Then, the swelling ratio (SR) of samples was calculated by

$$\text{SR}\left(\%\right)=\left({\text{W}}_{\text{t}}-{\text{W}}_{0}/{\text{W}}_{0}\right)\times 100\%$$ (2)

where W_t_ is the weight of the swollen sample at time t and W_0_ is the initial weight of the sample.

### 2.7 *In Vitro* Drug Release Analysis

The *in vitro* drug release profiles of samples were determined by the dialysis bag method. In brief, 0.2 g of the sample was loaded into a 3,500–5,000 Da cut-off dialysis bag and then dipped into the media containing different pHs (6.5 and 7.4) at 37°C under continuous stirring (300 rpm) for 72 h. At various time intervals, 1 ml of release medium was picked up for the analysis, and an equal volume of fresh buffer was added into the device to maintain a constant volume. The content of released drug was calculated by measuring its absorbance at a wavelength of 265 nm on using a UV-Vis spectrophotometer after the release medium was filtered through a 0.2 μm syringe filter. All the experiments were done in triplicate.

### 2.8 *In Vitro* Evaluation of Skin Permeation

To evaluate the *in vitro* skin permeation of chitosan**–**ibuprofen aerogels, the pig skin obtained from a local butcher was cleaned removing the fats and connective tissues and then put into hot water for 1 min. The skin samples were cut into squared samples (2.0 × 2.0 cm^2^) and immersed in phosphate buffer at different pHs (pH 7.4 or pH 6.5) for 2 h before use. Skin samples were mounted on a Franz diffusion cell with the epidermal side facing the receptor compartment and the stratum corneum facing the donor compartment containing 30 mg of chitosan**–**ibuprofen aerogels. The receptor compartment containing 20 ml of phosphate buffer was put into hot water at 37 ± 0.5°C under continuous stirring with magnetic fleas. 1 ml sample was withdrawn from the receptor compartment at predetermined time intervals, analyzed for ibuprofen content using a UV-Vis spectrophotometer after filtration using a 0.45 mm filter (Sartorius, Germany), and replaced with an equal volume of phosphate buffer to maintain sink conditions.

### 2.9 Mechanism of Ibuprofen Release Through Pig Skin

To understand well the release mechanisms of ibuprofen from chitosan**–**ibuprofen aerogels, four mathematical models including zero-order kinetic, first-order kinetic, Higuchi, and Hixson–Crowell mathematical models were established, and the results were used to evaluate the mechanism of ibuprofen release according to the degree of fitness into the mathematical models. The equations were analyzed as follows.

Zero-order kinetic model:

$$Q_{t}=\;K_{0}t,$$ (3)

First-order kinetic model:

$$In\left(1-Q_{t}\right)=-k_{1}\times t.$$ (4)

Higuchi model:

$$Q_{t}=K_{2}\times t^{1/2}.$$ (5)

Hixson–Crowell model:

$$1-{\left(1-Q_{t}\right)}^{1/3}=K_{3}\times t$$ (6)

In Eqs. 3–6, *Q*_*t*_ is the amount of ibuprofen release from the chitosan–ibuprofen nanoconjugate at time t and *K*_*0*_, *K*_*1*_, *K*_*2*_, and *K*_3_ are the release-rate constants.

## 3 Results and Discussion

### 3.1 Preparation of Chitosan**–**Ibuprofen Aerogels

**FIGURE 1:**
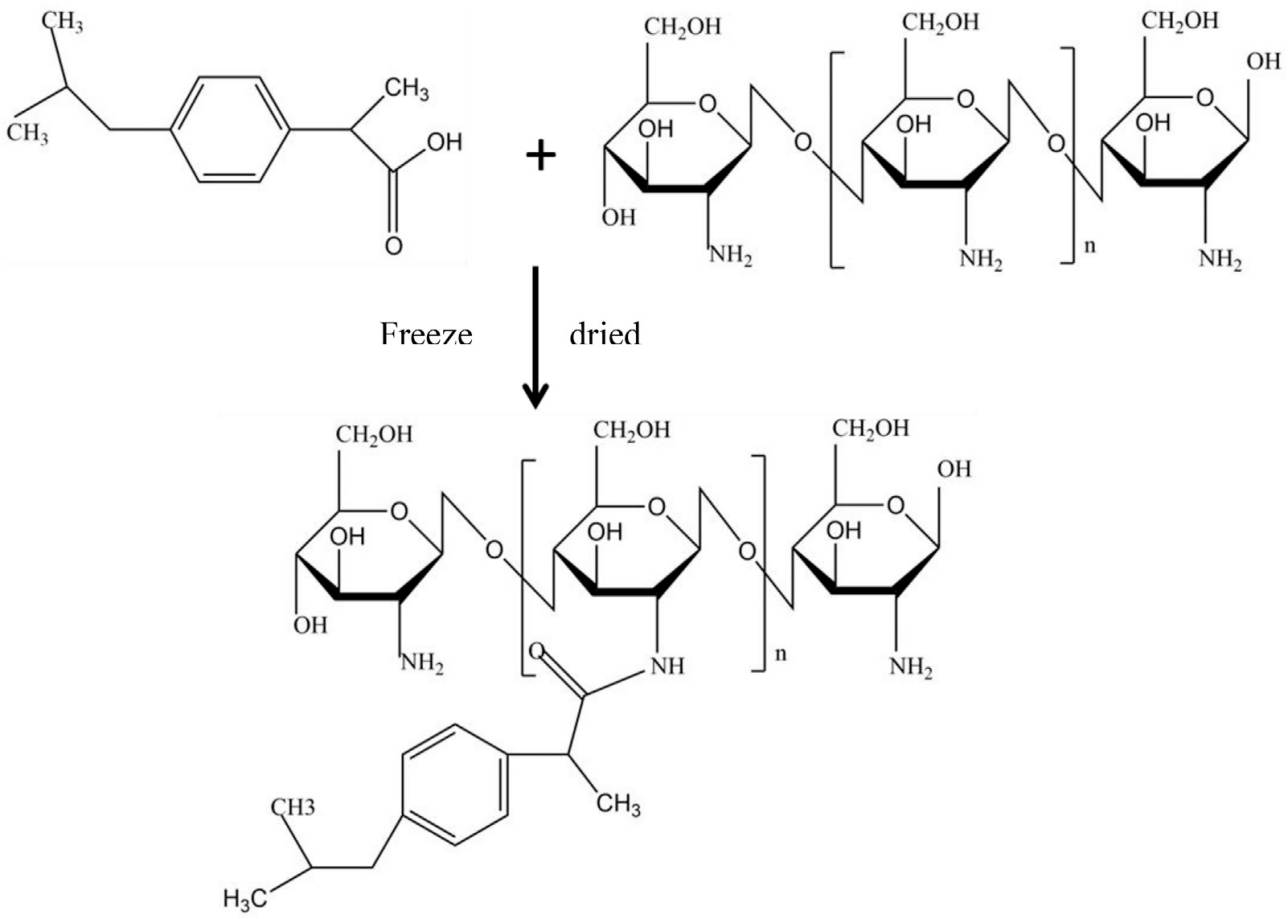
Schematic illustration of the synthetic routes of chitosan–ibuprofen aerogels.

The chitosan**–**ibuprofen aerogels were synthesized by a two-step procedure, and its illustration is shown in Figure 1. Chitosan was dissolved in 10 ml of 1% glacial acetic acid at 45°C, and 1 g of ibuprofen was solubilized in 10 ml of 0.1M NaOH solution. Then, the two obtained solutions were made up to 25 ml with distilled water, separately. Finally, the ibuprofen solution was added into the chitosan solution drop by drop and allowed to react for 2 h under continuous stirring at 45°C. The colloidal dispersion was vacuum-filtrated by a water-circulation multifunction vacuum pump with a vacuum filter holder. Then, they were kept in a refrigerator at −4°C and used for lyophilizing at −20°C under vacuum to obtain the target sample.

### 3.2 Characterization of Chitosan–Ibuprofen Aerogels

**FIGURE 2:**
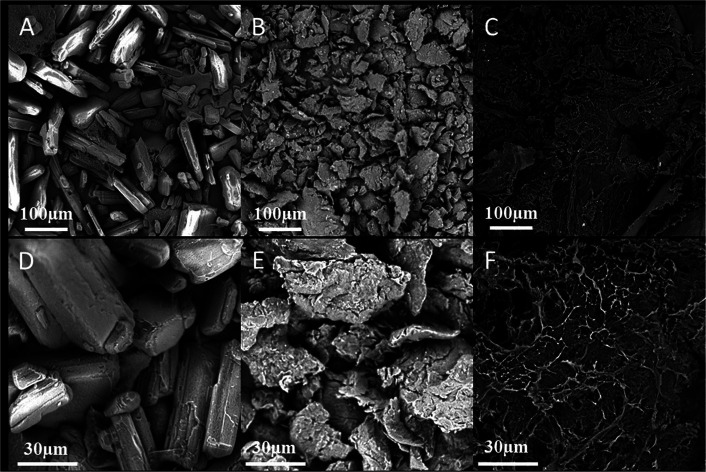
FE-SEM images of pure ibuprofen **(A, D)**, chitosan powder **(B, E)**, and chitosan–ibuprofen aerogels **(C, F)**.

The surface morphology of chitosan, ibuprofen, and chitosan–ibuprofen aerogels was scrutinized by scanning electron microscopy (SEM). As shown in Figures 2A,D, the pure ibuprofen exhibited a rod-like shape with the particle size of about 100∼200 µm. The photomicrographs of chitosan showed flaky shape with a thickness of 1∼2 µm (Figures 2B,E). In sharp contrast, in the presence of ibuprofen, the flaky shape characteristic of chitosan was not changed, but the thickness was decreased significantly and a lot of pores were found on flaky shape (Figures 2E,F), which may have resulted from the interaction between chitosan and ibuprofen.

**FIGURE 3:**
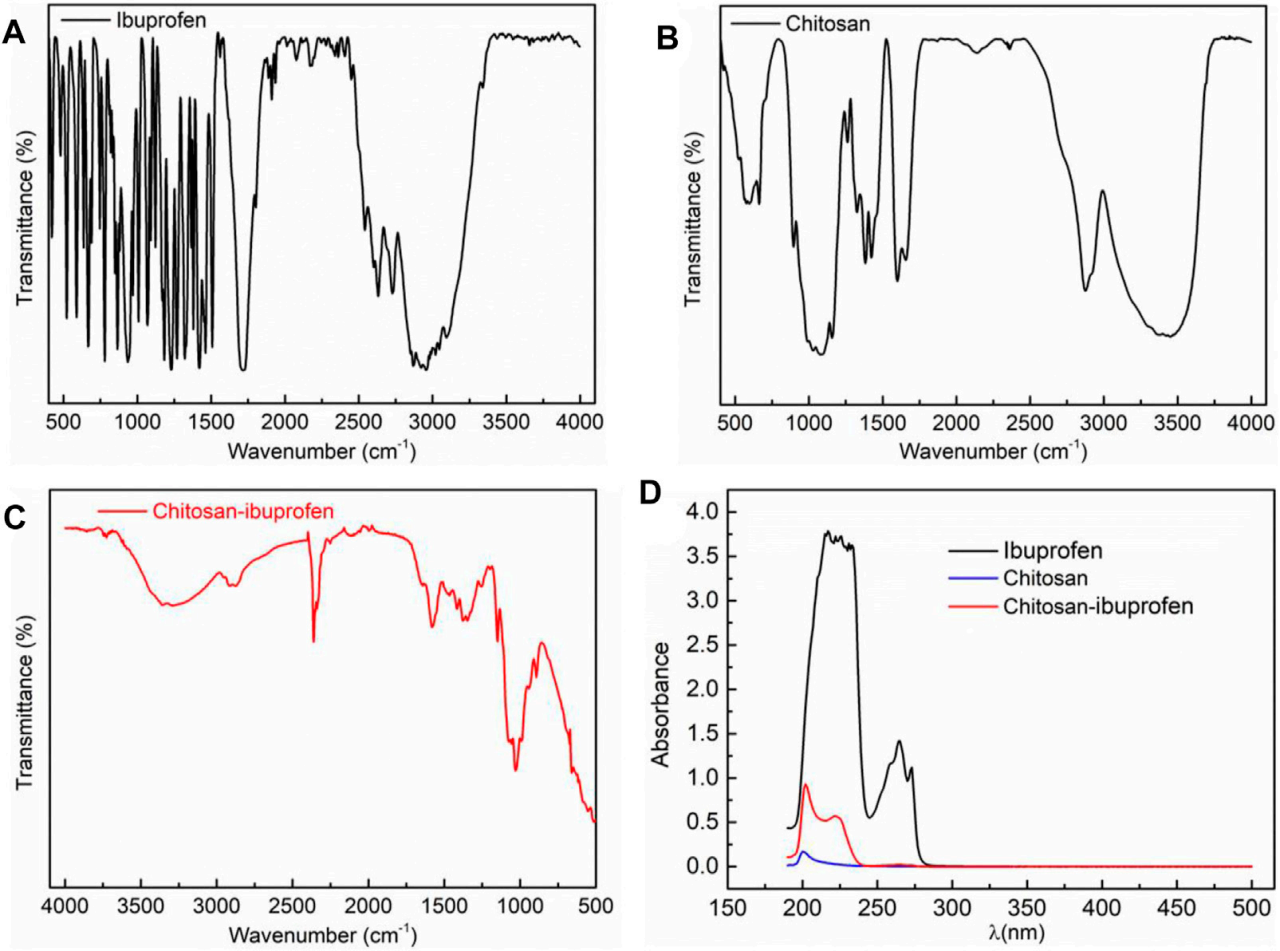
FTIR spectra of ibuprofen **(A)**, chitosan **(B)**, and chitosan–ibuprofen aerogels **(C)**; UV-Vis spectra of ibuprofen, chitosan, and chitosan–ibuprofen aerogels **(D)**.

The FTIR spectra of chitosan, pure ibuprofen, and chitosan–ibuprofen aerogels were determined, and the interaction of ibuprofen and chitosan was verified. As shown in Figure 3A, the ibuprofen spectrum exhibited characteristic bands at 3,096 cm^−1^, 1710 cm^−1^, 1,508 cm^−1^, and 1,229 cm^−1^ attributed to the -OH stretch, the asymmetrical wag from the carbonyl group (C=O), the aromatic ring vibration (C=C), and C-O stretching, respectively. The spectra of chitosan showed major characteristic bands of –O-H stretching vibrations at around 3,439 cm^−1^ and –C-H stretching vibrations at around 2,873 cm^−1^. The absorption bands at 1,656 cm^−1^ are amide I, and the absorption bands at 1,599 cm^−1^ and 1,378 cm^−1^ correspond to the vibration of the protonated amine group and amide III of chitosan, respectively. In addition, the absorption bands at 1,157 cm^−1^, 1,080 cm^−1^, and 1,028 cm^−1^ are characteristic of vibration of the CO group in its saccharide structure (Abioye et al., 2015) (Figure 3B). In sharp contrast, the FTIR spectra of chitosan–ibuprofen aerogels exhibited no significant difference from those of chitosan, but the stretching vibration peaks of –NH_2_ and –OH were significantly shifted to low wavenumbers corresponding to the strong intermolecular hydrogen bonding between chitosan and ibuprofen (Xu et al., 2020; Li et al., 2021) (Figure 3C). It is suggested that the ibuprofen molecule caused a change in the symmetry of chitosan in the nanoconjugate because of the electrostatic interaction and hydrogen bonding between the protonated amino group of chitosan and the carboxylic group of ibuprofen.

The UV-Vis spectra of ibuprofen, chitosan, and chitosan–ibuprofen aerogels were determined, and the results are shown in Figure 3D. In Figure 3D, we can see that ibuprofen showed an absorption peak at 225 and 265 nm and chitosan showed absorption peaks at around 200 nm. Compared to chitosan, the chitosan–ibuprofen aerogels showed two absorption peaks at about 202 and 225 nm, and the maximum absorption peak had a red shift phenomenon, which shifted to the long-wave direction by about 2 nm. This red shift may correspond to the transition between N-π * and π-π * during the copolymerization reaction of chitosan and ibuprofen. In addition, we can see from Figure 3D the intensity of the absorption peak at 202 nm is hugely increased. This result is consistent with the FTIR result.

**FIGURE 4:**
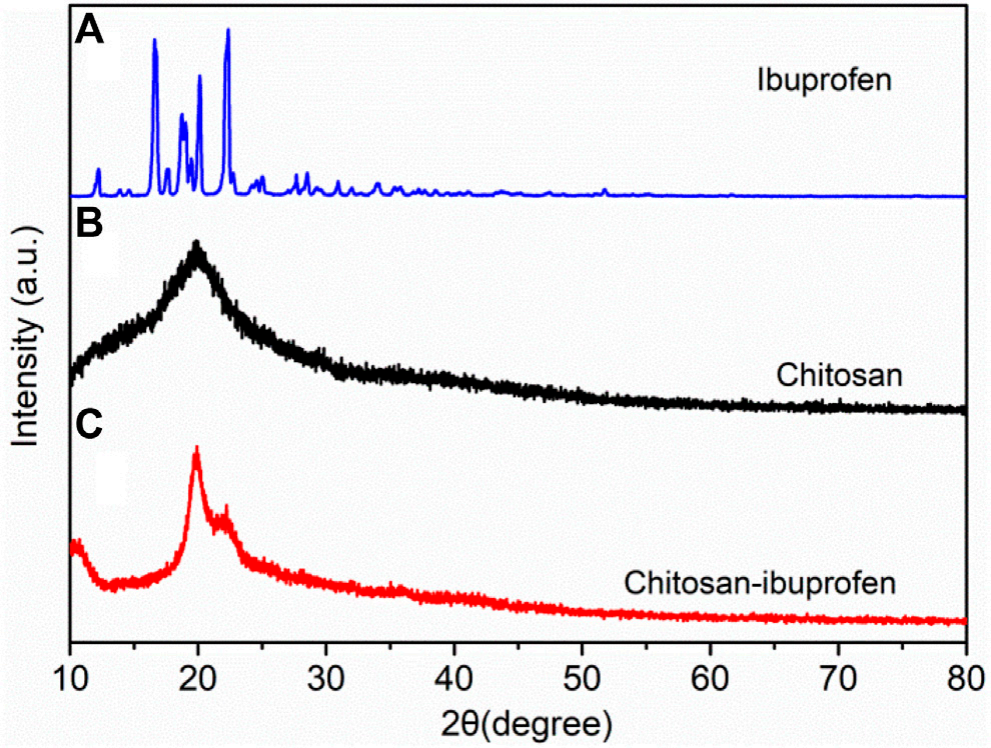
XRD patterns of ibuprofen **(A)**, chitosan **(B)**, and chitosan–ibuprofen aerogels **(C)**.

The diffraction patterns of ibuprofen, chitosan, and chitosan–ibuprofen aerogels were detected by the XRD technique. The XRD pattern of ibuprofen showed a characteristic peak of crystalline compounds (Figure 4A). The diffractogram of chitosan showed a broad peak at around 20°, corresponding to the (101) crystallographic planes, which indicates the amorphous structure of chitosan (Figure 4B). Compared with that of chitosan, the crystallinity of chitosan–ibuprofen aerogels has changed, which is attributed to the ionic interaction between the protonated amino group of chitosan and the deprotonated carboxyl group of ibuprofen (Figure 4C).

### 3.3 Thermal Behavior of Chitosan–Ibuprofen Nanoconjugate

**FIGURE 5:**
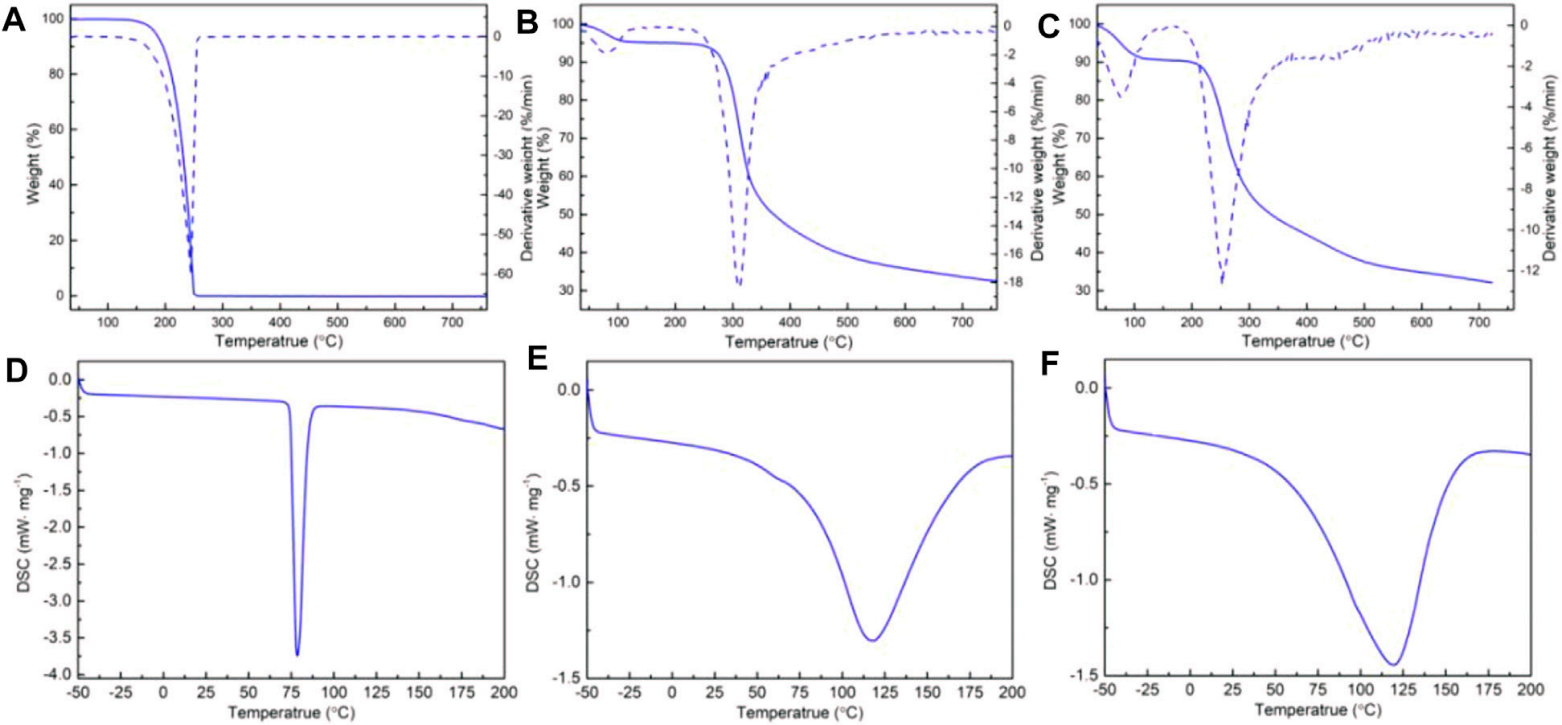
DSC thermograms of pure ibuprofen **(A)**, chitosan powder **(B)**, and chitosan–ibuprofen aerogels **(C)**; TGA thermograms of pure ibuprofen **(D)**, chitosan powder **(E)**, and chitosan–ibuprofen aerogels **(F)**.

**FIGURE 6:**
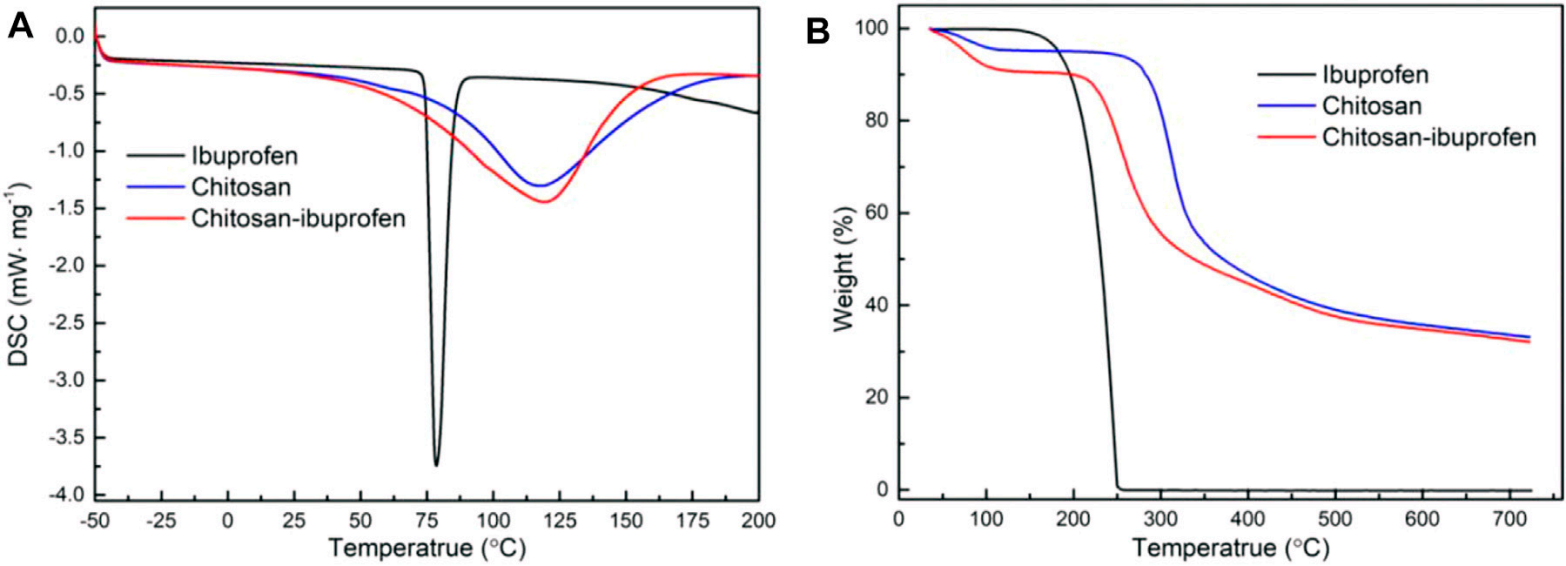
DSC thermograms **(A)** and TGA thermograms **(B)** of pure ibuprofen, chitosan, and chitosan–ibuprofen aerogels.

The thermo-analytical characteristics of chitosan, ibuprofen, and chitosan–ibuprofen aerogels were performed by means of thermogravimetric analysis (TGA) and differential scanning calorimetry (DSC). As shown in Figure 5D, a well-defined melting peak of ibuprofen was observed at about 81°C, there was an endothermic peak attributed to melting of ibuprofen, and no mass change was observed in the TGA thermogram (Figure 5A). However, in the TGA curve, ibuprofen exhibited a mass loss of about 99.75% in the range of 200∼240°C corresponding to the decomposition of ibuprofen. In the DSC thermogram of chitosan, we can observe a broad endothermic peak at 120°C owing to the loss of moisture content in the polysaccharide backbone, which is consistent with the mass loss of about 4.5% in the TGA curve (Figure 5E). In addition, a larger mass loss was about 50.15% at 311.9°C in TGA curves of chitosan, which corresponded to the decomposition of chitosan (Figure 5B). In the DSC curve of chitosan–ibuprofen aerogels, an endothermic peak was observed at 119°C which is lower than that of chitosan, indicating the decrease of water-holding capacity, which is attributed to the interaction of chitosan and ibuprofen (Figure 5F). In the TGA curve, chitosan–ibuprofen aerogels exhibited a mass loss of about 41.68% at 252.9°C attributed to the degradation of chitosan–ibuprofen. The faster mass loss rate (Figure 5C) and the increased onset of melting suggest the thermal stability and amorphous state of new chitosan–ibuprofen aerogels (Figure 5E). In addition, from Figure 6A, it can be seen that the DSC thermograms of the nanoconjugate exhibited a lower peak than that of chitosan, and the endothermic melting peak of pure ibuprofen does not appear, suggesting the interaction between chitosan and ibuprofen. Compared with chitosan, the chitosan–ibuprofen aerogels showed a lower weight loss in the temperature range from 350 to 600°C. It also suggests the chitosan–ibuprofen aerogels have a higher degradation temperature than chitosan (Figure 6B). These changes of thermal behavior of chitosan–ibuprofen aerogels are attributed to the interaction between ibuprofen and chitosan, and this result is in accordance with the FTIR result.

### 3.4 Swelling Degree Analysis

**FIGURE 7:**
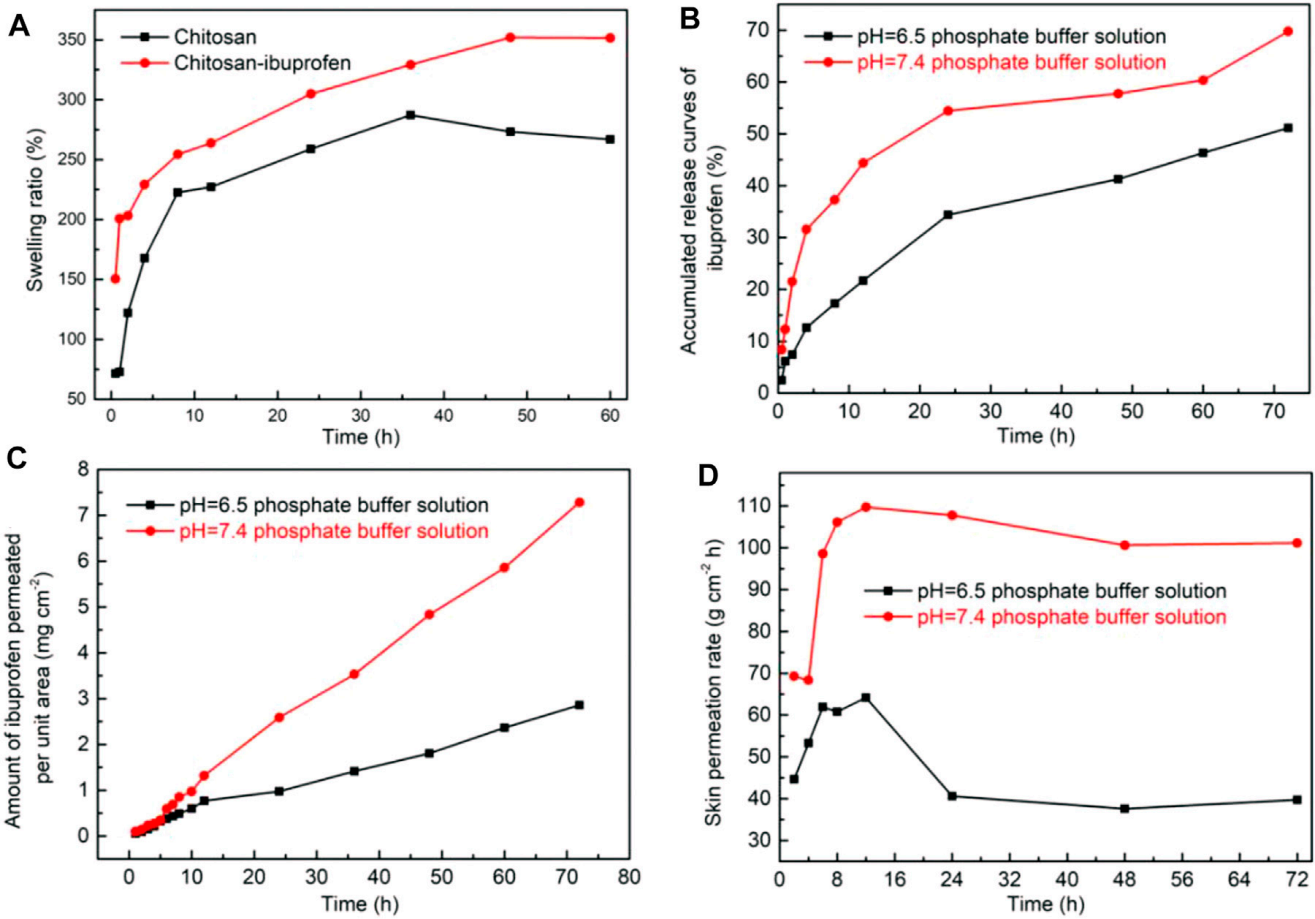
Swelling equilibrium curves of chitosan and chitosan–ibuprofen aerogels **(A)**; *in vitro* release curves of chitosan–ibuprofen aerogels in pH 7.4 and pH 6.5 phosphate buffer solutions **(B)**; *in vitro* release of ibuprofen from chitosan–ibuprofen aerogels through porcine skin **(C)**; skin permeation rate of ibuprofen in different pH phosphate buffer solutions **(D)**.

The swelling ratios of chitosan and chitosan–ibuprofen aerogels were determined in distilled water at 37°C, and the results are presented in Figure 7A. Chitosan and chitosan–ibuprofen aerogels showed an increased swelling ratio in 36 h, and the swelling of chitosan–ibuprofen aerogels was higher than that of chitosan. The increase in swelling of the two samples suggests the better water uptake capacity. After 36 h, the swelling of chitosan showed a decreased trend owing to dissolution or degradation of chitosan. In contrast, the swelling of chitosan–ibuprofen aerogels still increased to the maximum of 352.8%, indicating an excellent swelling ratio corresponding to the interaction between chitosan and ibuprofen and their nanostructure, which increases the hydrophilic nature and the diffusion of the water molecule.

### 3.5 *In Vitro* Drug Release Analysis

The results of the *in vitro* release of ibuprofen by chitosan–ibuprofen aerogels in different pHs of medium over time are shown in Figure 7B. As seen in Figure 7B, the accumulated release rate of ibuprofen was increased slowly without any sign of plateau during 72 h in pH 6.5 and pH 7.4 PBS solutions, which corresponded to the electrostatic and hydrophobic interactions between chitosan and ibuprofen suggesting the potential of the aerogels to control the release of ibuprofen. The release of ibuprofen reaches a maximum of 69.99% in pH 7.4 PBS solution and 51.0% in pH 6.5 PBS solution. The excellent release behavior corresponded to the swelling and degradation behaviors of chitosan–ibuprofen aerogels, which provided the channels for drug diffusion. In addition, we can observe that the accumulated release rate of ibuprofen in pH 7.4 PBS solution is markedly higher than that in pH 6.5 PBS solution, which may be due to the swelling and degradation behaviors of chitosan–ibuprofen aerogels and the better solubility of ibuprofen in pH 7.4 PBS solution.

### 3.6 *Ex Vivo* Skin Permeation and Drug Release Mechanism Studies

*Ex vivo* skin permeation and release mechanism of ibuprofen from chitosan–ibuprofen aerogels through porcine skin were studied, and the results are shown in Figure 7C and Table 1. In Figure 7C, we can see that the cumulative release amounts of ibuprofen permeated through porcine skin at different pHs of PBS solution were increased slowly in 72 h and exhibited excellent permeability corresponding to the bioadhesive characteristic of chitosan, which enhanced the diffusion of ibuprofen through porcine skin by disrupting the lipid bilayer of the skin. The loose structure resulted from the swelling and degradation behaviors of chitosan–ibuprofen aerogels also can improve the permeability of drug through pig skin. We can also observe that the trend of drug release from chitosan–ibuprofen aerogels was slow without any sign of plateau, which is attributed to the electrostatic and hydrophobic interactions between the drug and the polymer. Furthermore, we can observe that the permeation rate of ibuprofen was increased first and then decreased corresponding to the reduced concentration of ibuprofen (Figure 7D). In addition, the drug amount of drug released and the permeation rate of drug in pH 7.4 PBS were higher than those in pH 6.5 PBS due to the relatively loose structure resulted from the swelling or degradation of chitosan–ibuprofen aerogels in pH 7.4 PBS, which reduced the higher resistance to drug release.

**TABLE 1:** Permeation parameters and release mechanisms of ibuprofen from chitosan–ibuprofen aerogels derived from different mathematical models of drug release kinetics.

Mathematical models	Zero-order kinetics	First-order kinetics	Higuchi model	Hixson–Crowell model
Equation	Q_t_ = K_0_×t	ln (1- Q_t_) = -K_1_×t	Q_t_ = K_2_×t	1-(1- Q_t_)_1/3_ = K_2_×t
Mechanism of release	Constant rate of release	Diffusion (Fick’s first law)	Diffusion and permeability	Erosion release
Parameters	K_0_, R^2^	K_1_, R^2^	K_2_, R^2^	K_3_, R^2^
Chitosan–ibuprofen (pH = 7.4 PBS)	0.10, 0.9989	0.18, -0.1468	0.64, 0.8231	0.04, 0.9981
Chitosan–ibuprofen (pH = 6.5 PBS)	0.04, 0.9888	0.10, 0.4087	0.26, 0.8770	0.02, 0.9942

### 3.7 Release Mechanism of Ibuprofen Release Through Pig Skin

In order to clarify the mechanisms of drug release through pig skin, the *ex vivo* release data of chitosan–ibuprofen aerogels were fitted into mathematical models, zero-order, first-order, Higuchi, and Hixson–Crowell models, as shown in Table 1. The chitosan–ibuprofen aerogels fitted best into zero-order kinetics (R^2^ = 0.9989, 0.9888), which indicated slow release of the same amount of drug per unit time at a constant rate. In contrast, the chitosan–ibuprofen aerogels also fitted well into the Hixson–Crowell model in pH 6.5 PBS (R^2^ = 0.9942), indicating the dissolution rate of discrete particles limited the rate of ibuprofen release. From the foregoing results, we can conclude that drug release from the chitosan–ibuprofen aerogels through the pig skin barrier included four periods: drug diffusion, drug partition, polymer swelling, and matrix erosion, and these enhanced the controlled extended release profile (Figure 7A). The drug release phases correspond to the increased stability and swelling rate of chitosan–ibuprofen aerogels, which resulted in a sustained drug release through pig skin.

## 4 Conclusion

Chitosan–ibuprofen aerogels can be readily synthesized by a simple method. Our study found that the chitosan–ibuprofen aerogels showed a nanopore morphology structure, and the introduction of ibuprofen changed the crystallinity of chitosan attributing to the electrostatic and hydrophobic interactions between the drug and the polymer. In addition, the chitosan–ibuprofen aerogels have an excellent swelling ratio and higher thermal stability. From the results of *in vitro* release, permeability of drug through pig skin, and mechanisms of drug release, we can conclude that polymer–drug aerogels are a potential material for controlled *in vitro* release and transdermal release of ibuprofen. Our study highlights the great potential of chitosan as a component phase in the transdermal drug release system to control the release of ibuprofen.

## Data Availability

The original contributions presented in the study are included in the article/supplementary materials, and further inquiries can be directed to the corresponding author.
